# Controlled trial of decision support for men with early-stage prostate cancer: brief research report of effects on patient knowledge

**DOI:** 10.3389/fruro.2023.1127089

**Published:** 2023-05-17

**Authors:** Jeffrey K. Belkora, Jeanette M. Broering, John Neuhaus, Ali Zargham, Tia Weinberg, John S. Witte, Stacey A. Kenfield, Erin L. Van Blarigan, Matthew R. Cooperberg, Peter R. Carroll, June M. Chan

**Affiliations:** ^1^ Institute for Health Policy Studies, University of California, San Francisco, San Francisco, CA, United States; ^2^ Department of Urology, University of California, San Francisco, San Francisco, CA, United States; ^3^ Department of Surgery, University of California, San Francisco, San Francisco, CA, United States; ^4^ Department of Epidemiology & Biostatistics, University of California, San Francisco, San Francisco, CA, United States

**Keywords:** patient education, personalized health care, prostate cancer, active surveillance, health coaching, decision aid, decision support, service learning internships and co-ops

## Abstract

**Introduction:**

A single-arm pre-post pilot study in an academic setting found that pre-consultation decision support was associated with improved patient knowledge among men with early-stage prostate cancer. We now report on exploratory analyses from a controlled study featuring patients from both academic and community settings.

**Methods:**

We enrolled 58 men to usual care and 61 men to the intervention. We evaluated whether the intervention was associated with patients answering key knowledge items correctly just before their urology visit.

**Results:**

Just prior to the urology visit, 39/56 or 70% in the intervention group replied correctly to key knowledge items, compared to 31/55 or 56% in the usual care group (p=0.15). At baseline, the intervention group started with 42/60 or 70% correct and the usual care group started with 28/56 or 50% (p=0.03). This imbalance at baseline created a ceiling effect: more men in the usual care group had room to improve on their knowledge scores. Indeed, seven men moved from incorrect to correct in the usual care group, versus 5 in the intervention group; and five men in the intervention group moved from correct to incorrect versus 3 in the usual care group (p=0.44).

**Discussion:**

In addition to small sample size, reasons for the null findings may include clustering of highly educated participants at the academic site combined with over-representation of academic site participants in the intervention group. We confirmed, from the pilot study, the feasibility of using pre-health student interns as health coaches. Future research should explore whether increasing adoption of telehealth will improve the feasibility of delivering pre-visit decision support in community settings.

## 1 Introduction

People diagnosed with serious illness generally are at risk for both under-treatment and over-treatment relative to their personal priorities, disease risk characteristics, and the best available medical evidence (1–3). This problem is salient in the case of low-risk prostate cancer patients, who are often over-treated with surgery or radiation relative to what they say they would have preferred if fully informed about all the options and outcomes, including the risks and benefits of active surveillance.

Decision support interventions tailored to specific clinical crossroads have increased patient self-efficacy, knowledge, question-asking, and satisfaction; and decreased decisional conflict, regret, anxiety, and distress (1, 4–16). More informed and involved patients tend to pursue less invasive treatment options than less informed and involved patients (17, 18).

However, in the area of prostate cancer, decision support interventions have not provided personalized risk estimates to patients. The need for personalized risk estimates arises from the potential for an initially low-risk prostate cancer to be reclassified over time as higher-risk. Many patients and physicians avert the risk of reclassification by erring on the side of active treatment (surgery or radiation) from the start. Another reasonable strategy would be to choose active surveillance (implying close monitoring) and switch to active treatment only in those cases where the patient’s risk increased over time.

To support fully informed decision making by patients, our team developed a comprehensive decision support intervention (DSI). Our DSI prompts patients to review their individual risk and other educational materials, as well as make a list of questions in writing for their physicians (6). We also trained a service learning workforce to deliver the intervention remotely, by internet and telephone, in a way that does not require clinics to modify their practices, and that leverages student interns receiving academic credit as part of their academic training (6).

We hypothesized that delivering decision support would increase patient knowledge, and this proximal outcome would ultimately increase the proportion of men making informed decisions regarding their selection of active surveillance, surgery, or radiation for low-risk prostate cancer. Our initial pilot study in an academic setting found our intervention feasible and effective in informing men of two key facts prior to their first urology visit upon diagnosis with low-risk prostate cancer (6). Because our intervention has novel elements, including pre-visit provision of personalized risk estimates delivered by student health coaches, we now report on a study including patients at four community sites.

## 2 Methods

### 2.1 Context, setting, population, study design, and sample

We set out to recruit men with low-risk prostate cancer at four community sites. We chose a convenience sample of four community sites from active enrolling sites (2016-2017) of the Cancer of the Prostate Strategic Urologic Research Endeavor (CaPSURE) study (19), representing different geographic regions of the United States. We designed a four-site, site-randomized, two-period, cluster-crossover study and planned to enroll 160 participants, allowing for attrition (12) and non-response (12), leaving 136 fully evaluable participants (ClinicalTrials.gov registry NCT03397160). We randomized sites in pairs (blocks size 2) to start period 1 as usual care or intervention, with the starting allocation also randomized. We planned to cross over each site to intervention or usual care in period 2 after they met *a priori* accrual goals (i.e., N~40 per site, 20 men per period). We budgeted funds for a research coordinator at each site to screen men into the following eligibility criteria and then offer enrollment and guide participation in the trial.

Men were considered eligible for this study if they were diagnosed with localized low-risk prostate cancer defined as: diagnostic PSA <= 15 ng/ml; clinical stage cT1/2, N0, M0; and Gleason sum 2-6 (or Gleason score 3 + 4 with <=33% cores positive for adenocarcinoma) based on a diagnostic biopsy with a minimum of 10 cores. Additionally, men had to have not received or already decided on any treatment for prostate cancer, have had no prior use of 5-alpha reductase inhibitors with 6 months of the biopsy (criteria intended for ancillary correlative science study), have English language proficiency and ability to sign an informed consent form; and be considered candidates for active surveillance at their institution by their treating urologist.

In practice, we encountered problems implementing the study: turnover of staff at community sites; fewer than expected patients met the eligibility criteria; and we depleted time and budget on screening out large numbers of ineligible patients. Therefore, in July 2018, after 9 months of slow enrollment, we added the University of California, San Francisco (UCSF), which was the site of the prior academically-based pilot study. Our rationale was that, while our site-randomized crossover trial was proving infeasible, we could still seek to replicate our uncontrolled pilot study results in a controlled study. In addition, given depletion of original funding, the slow accrual, and the onset of the COVID-19 pandemic, we had to close the study prior to reaching the original accrual goal of 160, after accruing 119 participants. We now report briefly on our findings, which should be considered exploratory, and illustrate lessons learned from attempting to translate a pre-visit intervention for low-risk patients from an academic pilot study to community settings.

### 2.2 Outcomes, measures, and instruments

#### 2.2.1 Primary outcome: knowledge of two key facts

The primary endpoint was the proportion of patients who responded accurately to two survey items from the Prostate Cancer Decision Quality Instrument (20), administered at the clinic by a study coordinator just before the consultation with the urologist. The two items assessed whether, among men diagnosed with early-stage prostate cancer, most will die of prostate cancer; about half will die of prostate cancer; or most will die of something else (answer: most will die of something else); and whether waiting three months to make a treatment decision will affect their survival a lot; somewhat; or a little or not at all (answer: a little or not at all).

Knowledge of these key facts is an important endpoint because patients often associate the word cancer with high mortality risks, and urgency to act. Patients also often associate more invasive treatment with greater benefit.

In the case of early-stage, low-risk prostate cancer, patients would ideally recognize that they are more likely to die of other causes than prostate cancer and that most can safely take three months to make decisions. This knowledge potentially opens patients up to consideration of active surveillance along with surgery and radiation therapies.

Our intervention aimed to educate patients about their low mortality risk and the time available for decision-making after their cancer diagnosis and before they saw their urologist for primary treatment counseling. Therefore, we assessed this endpoint just prior to the first urology appointment after a biopsy confirmed early-stage low-risk cancer. We compared results at this timepoint between the usual care and intervention groups. We also assessed the knowledge endpoint at baseline to evaluate within-subject changes in knowledge; and repeated the knowledge assessment after the consultation with the urologist.

### 2.3 Intervention

As reported earlier (6), we developed a decision aid to present patients with the following estimates: survival and quality of life outcomes from the Cancer of the Prostate Strategic Urologic Research Endeavor (CaPSURE) outcomes database for patients who underwent active surveillance, surgery, or radiation; and risk of reclassification (“upstaging” or “upgrading”) based on demographic, clinical, and pathologic parameters (i.e., age, prostate volume, prostate tumor grade, percent positive cores from biopsy, PSA) using a model developed at UCSF and tested using UCSF and CaPSURE data (21).

After reviewing the decision aid, either *via* a secure website, mailed screenshots, or emailed screenshots, intervention group patients spoke with a pre-health student acting as a coach. Per the pilot study intervention, we trained four coaches to be neutral and non-directive in reviewing the contents of the decision aid and in helping the study participants make a list of questions (4). The coach reviewed the decision aid screen by screen with the patient, reviewed built-in help text with the patient when appropriate, and wrote down (without answering) each patient’s questions for the physician. The coach saved a word-processed document with the patient’s questions for the physician, and sent that file to the site study coordinator, who printed copies for the patient, family, and physician and made those copies available to all parties at the time of the clinic visit. We assured fidelity of the coaches to the intervention by recording and reviewing role-plays with simulated patients.

For the low-risk patients eligible for our study, survival projections were relatively high, and risk of reclassification was relatively low. We expected the intervention to communicate to patients that they were more likely to die of causes other than prostate cancer and that they had time to make decisions.

### 2.4 Analysis plan

The primary statistical analysis compared, for assessments made just before the patient consultation with their urologist, the proportion of patients in intervention versus control groups responding correctly to both key knowledge items; and the proportion of patients whose answers changed from at least one incorrect at baseline to both correct before the urologist visit. We used the chi-square test (and p-values) to compare proportions across groups; and conditional logistic regression (and 95% confidence intervals (CI)) for comparing the changes within subjects. A threshold of 0.05 was used to determine statistical significance. We excluded from each analysis any patients who were missing a required response.

## 3 Results

### 3.1 Study sample

**Figure 1:**
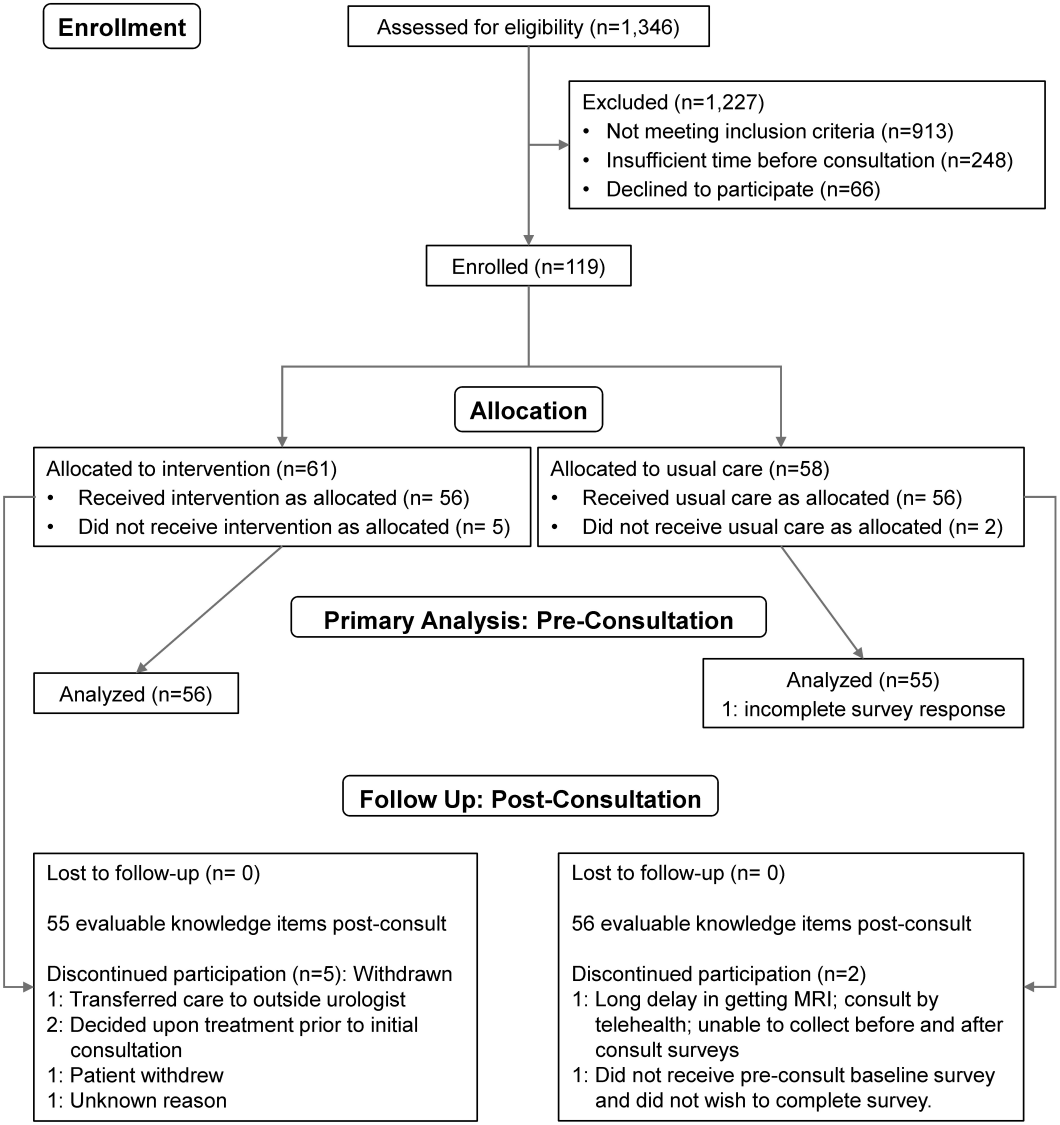
Diagram showing the flow of participants through the study.

Among the four sites that enrolled patients, we screened 1,346 patients, of whom 913 (68%) did not meet our study’s eligibility criteria. The main reasons for ineligibility were for not meeting clinical criteria of low-risk disease (e.g., ~40% of 855 men screened did not meet the Gleason grade criteria described above). This left 433 potentially eligible patients. Research coordinators were unable to reach 249 of these patients ahead of their appointment in time to offer them participation, leaving 185 patients who were both eligible and available. Of these, 66 (36%) declined and 119 (64%) enrolled. Study participants received the intervention or usual care based on the study phase their site was in at the time of their enrollment. After sites I and II accrued participants very slowly, we added our academic site in the next block (III, IV). When site IV failed to accrue participants, we replaced it with site V, which also accrued slowly. In the original design, all sites were to cross over after accruing their share of patients; in practice, only sites II and III accrued sufficiently to cross over and contribute to a controlled comparison. See **Figure 1** and **Table 1** for details of the participant allocation and ascertainment by site. Screening began 10/16/2017 and concluded on 3/20/2020. **Table 2** summarizes the sample demographics. Given these deviations from our original enrollment plans, the intervention and control groups were not balanced between the academic site and the community sites, and not balanced in terms of participant characteristics (most notably education level), possibly due to clustering of participants in the different settings (**Tables 1**, **2**). While the slow accrual meant that we could not implement our original study design, for ethical reasons we report on our exploratory analyses of the data as collected.

**Table 1:** Summary of participant recruitment by site.

Start Date	Cross Date	Order of Phases	Site	SCR	INEL	EL	No time	Time	DEC	EN	INT	Usual Care
10/16/17	NA	Intervention, Usual Care	I (Community East)	156	119	37	13	24	16	8	8	0
11/1/17	4/27/19	Usual Care, Intervention	II (Community Midwest)	451	281	170	120	50	23	27	4	23
7/13/18	2/25/19	Usual Care, Intervention	III (Academic)	602	426	176	75	101	22	79	44	35
7/16/18	NA	Intervention, Usual Care	IV (Community West)	15	9	6	6	0	0	0	0	0
8/2/19	NA	Intervention, Usual Care	V (Community West)	122	78	44	34	10	5	5	5	0
			TOTAL (%)	1,346	913 (68)	433 (32)	248/433 (57)	185/433 (43)	66/185 (36)	119/185 (64)	61/119 (51)	58/119 (49)

SCR, screened; INEL, ineligible; EL, eligible; No time = patients where there was not enough lead time before appointment to offer participation; Time = patients where there was sufficient lead time before the appointment to offer participation; DEC, declined; EN, Enrolled; INT, intervention; Usual Care, no intervention.

**Table 2:** Demographic characteristics of decision support versus usual care groups.

Characteristic	Intervention Group N = 61	Usual Care Group N = 58	Statistical Test *P* value
	Mean (SD)	Mean (SD)	
Age *(average age at diagnosis)*	62.9 (8.9)	62.5 (6.6)	0.78 (a)
Ethnicity	N (%)	N (%)	
Hispanic	1 (1.6)	5 (9.1)	0.08 (b)
Non-Hispanic	60 (98.4)	50 (90.9)	
Declined to report	–	3	
Race^b^			
American Indian/Alaska Native	1 (1.6)	1 (1.8)	0.60
Asian	5 (8.2)	1 (1.8)	
Black	1 (1.6)	1 (1.8)	
Native Hawaiian	–	1 (1.8)	
White	52 (85.3)	48 (87.3)	
More than one race	2 (3.3)	3 (5.5)	
Declined to report	–	3	
Education^b^			
Some high school	–	2 (3.8)	0.03
High school or GED	13 (21.7)	12 (22.6)	
College degree	17 (28.3)	25 (47.2)	
Graduate/professional degree	30 (50.0)	14 (26.4)	
Declined to report	1	5	
Work Status^b^			
Full-time	27 (45.8)	30 (52.6)	0.45
Part-time	3 (5.1)	5 (8.8)	
Retired	29 (49.2)	22 (38.6)	
Declined to report	3	1	
Income^b^ (annual household)			
$10-20K	2 (4.0)	1 (2.9)	0.63
$21-30K	1 (2.0)	–	
$31-50K	3 (6.0)	2 (5.7)	
$51-75K	4 (8.0)	7 (20.0)	
$76-100K	8 (16.0)	2 (5.7)	
$101-125K	2 (4.0)	2 (5.7)	
$126-150K	4 (8.0)	4 (11.4)	
>$150K	26 (52.0)	17 (48.6)	
Declined to report	11	23	

^(a)^ Calculated using two-sample t-test with equal variance.

^(b)^ Used Fisher’s exact test to accommodate small cell sizes in many of the demographic categorial comparisons. Participants who declined to report an attribute were treated as ‘missing data’ and were excluded from the Fisher’s exact test comparisons.

### 3.2 Exploratory analyses

At baseline, the intervention group started with 42/60 or 70% correct and the usual care group started with 28/56 or 50% correct. This difference was statistically significant (p=0.03). Before the consultation with their urologist, after intervention 39/56 or 70% in the intervention group answered correctly compared to 31/55 or 56% in usual care. The difference (our primary analysis) was not statistically significant (p=0.15). After the consultation with the urologist, 45/55 or 82% in the intervention group were correct, and 37/56 or 66% in the usual care group. The difference was not statistically significant (p=0.06).

Of the 10 men in each group who changed their responses between baseline and post-intervention assessment of knowledge, 5/10 (50%) moved from incorrect to correct in the intervention group versus 7/10 (70%) in the usual care group. This difference was not statistically significant (p=0.44).

## 4 Discussion

### 4.1 Discussion of primary outcome

The intervention group reported higher knowledge at all three time points: at baseline, immediately before, and immediately after consultation with the urologist. In this respect, this controlled study replicated the findings of the pilot study. However, the magnitude of the differences were smaller than in the pilot study (6), and our comparisons to usual care did not show statistically significant differences. One possible explanation is that at baseline, before any intervention, the intervention group answered 70% correct whereas the usual care group was 50% correct. This difference was likely due to imbalances in accrual by site type, and clustering of similar, highly educated participants at the academic site. Specifically, more intervention patients came from the academic site than community sites; and more participants at the academic site had higher levels of education. For example, 50% of the participants in the academic site had graduate or professional degrees, compared to 26% in the usual care site. The ensuing differences in intervention and control groups may have resulted from the difference in their starting knowledge scores, i.e. a ceiling effect. The intervention group had few participants with room to improve.

### 4.2 Lessons learned

After it became clear that our study would not accrue sufficient patients in its original community site crossover design, we added the academic site from the pilot study. This allowed us to conduct a controlled exploratory analysis, and use the data collected from community site patients who contributed their participation and deserved our best efforts to leverage their participation into insights. We learned several lessons that may be valuable for clinicians and researchers, summarized below.

#### 4.2.1 Cautionary lessons learned about the challenges of priming patients with education before visits to urologists

Our intervention attempted to screen and contact patients before they saw a urologist to discuss treatment options for a diagnosis of early-stage, low-risk prostate cancer. Priming patients for a visit should logically improve their participation (e.g. question-asking) and recall of information at the visit. Studies in academic settings have shown such effects, including in the domain of prostate cancer (8–10). At the time of the study, however, our community partners were more used to screening patients and interacting with them after their first in-person clinical visit, not before. This is an important distinction that clinicians and researchers should consider when translating pre-visit interventions from academic to community settings. In this study, the academic site was able to approach 101 out of 176 eligible patients (57%), whereas the community sites approached only 84 out of 257 eligible patients (33%). This is likely because academic sites build up a strong capacity to recruit patients to a variety of studies, including those that require interaction before any visit takes place.

Looking to the future, it may be that pre-visit interventions are more feasible in settings with more telehealth capabilities. Our study took place before the massive increase in telehealth surrounding COVID. Community settings are now embracing telehealth in the wake of the response to COVID-19. In addition, with the recent advances in use of electronic health records, future studies could leverage patient portals to screen for eligibility, offer electronic consenting, and deliver educational interventions.

#### 4.2.2 Cautionary lessons learned about the challenges of identifying and intervening with low-risk patients

Another insight from our study that may help future clinicians and researchers relates to low-risk patients. In the domain of prostate cancer, low-risk patients may seek or accept unnecessarily aggressive treatment which they would avoid if they fully understood their risk profile. Our screening process, looking for low-risk patients, yielded smaller than expected numbers of eligible patients. One explanation may be that clinician attitudes to diagnosing prostate cancer were evolving – i.e., clinicians became more concerned with over-diagnosing prostate cancer and were waiting for higher prostate-specific antigen test scores before conducting biopsies. More patients than expected became ineligible for our study because either their prostate-specific antigen scores or tumor Gleason scores exceeded our low-risk eligibility threshold by the time they had a biopsy and were ready to consider treatment. This raises the question of when and how to identify low-risk patients and educate them. Our intervention was novel in attempting to educate patients after a biopsy but before their appointment with a urologist to discuss treatment options. Perhaps educational interventions should be aimed even further upstream, at patients with rising prostate-specific antigen scores who are being followed to consider biopsies. Essentially these patients are starting with active surveillance and could begin to consider how they might respond given possible biopsy results. Here again, patient education for cohorts of patients that are still being seen in primary care will likely be easier with new advances in telehealth, including patient portals.

### 4.3 Strengths and limitations

The final study implementation was an academic and community based, unblinded, controlled trial that did not meet original accrual goals. This outcome left us susceptible to several biases. Our study may reflect sampling bias and clustering. Because sites followed a different diagnostic protocol, they had different lead times for study recruitment, which differentially affected study accrual rates across the sites, another form of sampling bias. Most participants came from an academic site where a potential ceiling effect in the primary outcome, knowledge, was more pronounced at baseline than at community sites. This may have been due to the imbalance in characteristics (notably, education level) of participants who clustered around academic versus community sites.

We enrolled higher numbers of usual care participants early in the study compared to intervention group participants, so the maturation (passage of time) bias was different in each group. We believe the passage of time was associated with changing diagnostic and treatment practices among clinicians and could have been associated with different levels of knowledge among study participants. Specifically, the intervention group participants may have started at a higher level of knowledge about their mortality risk simply because clinicians and the media were paying more attention over time to the need to avoid overdiagnosis and overtreatment among men with low-risk prostate cancer.

While our study was underpowered to detect differences between the groups, especially in the context of a ceiling effect in the intervention group, this experience offers a few valuable lessons. We replicated the feasibility, in our pilot study, of using undergraduate student interns as coaches. They earned academic credit through their participation in a service learning program, the Patient Support Corps (6). In course ratings, students expressed satisfaction with the service-learning experience. Using students as workforce extenders helped the study conserve budgetary resources while still providing personal attention to study participants. Students were able to coach patients by telephone while the patient looked at a decision aid over the internet.

In conclusion, we did not observe a statistically significant increase in patient knowledge with the implementation of a decision support intervention incorporating tailored risk education and health coaches. The breakdown in study design, resulting imbalances between groups, lack of sufficient statistical power, secular trends in prostate cancer screening and diagnosis, and ceiling effects in the primary outcome may partially explain these null findings.

## Data availability statement

The raw data supporting the conclusions of this article will be made available by the authors, without undue reservation.

## Ethics statement

The studies involving human participants were reviewed and approved by UCSF Human Research Protection Program Institutional Review Board. The patients/participants provided their written informed consent to participate in this study.

## Author contributions

The Author Contributions section is mandatory for all articles, including articles by sole authors. The Author Contributions statement must describe the contributions of individual authors referred to by their initials and, in doing so, all authors agree to be accountable for the content of the work. JKB: study conception and design, data analysis and interpretation, manuscript drafting and revisions. JMB: study conception and design, data collection, data analysis and interpretation, manuscript drafting and revisions. JN: study conception and design, data analysis and interpretation, manuscript review and revisions. AZ: study conception and design, data collection, manuscript review and revisions. TW: study conception and design, data collection, manuscript review and revisions. SK: study conception and design, manuscript review and revisions, JW: data analysis interpretation, manuscript review. EB: study conception and design, manuscript review and revisions. MC: study conception and design, manuscript review and revisions. PC: study conception and design, manuscript review and revisions. JC: study conception and design, data analysis and interpretation, manuscript review and revisions. All authors contributed to the article and approved the submitted version.
